# Temporal dynamics of mother–offspring relationships in Bigg's killer whales: opportunities for kin-directed help by post-reproductive females

**DOI:** 10.1098/rspb.2023.0139

**Published:** 2023-06-14

**Authors:** Mia Lybkær Kronborg Nielsen, Samuel Ellis, Michael N. Weiss, Jared R. Towers, Thomas Doniol-Valcroze, Daniel W. Franks, Michael A. Cant, Graeme M. Ellis, John K. B. Ford, Mark Malleson, Gary J. Sutton, Tasli J. H. Shaw, Kenneth C. Balcomb, David K. Ellifrit, Darren P. Croft

**Affiliations:** ^1^ Centre for Research in Animal Behaviour, University of Exeter, Exeter, UK; ^2^ Center for Whale Research, Friday Harbor, WA, USA; ^3^ Bay Cetology, Alert Bay, British Columbia, Canada; ^4^ Pacific Biological Station, Fisheries and Oceans Canada, British Columbia, Canada; ^5^ Department of Biology, University of York, York, UK; ^6^ Faculty of Environment, Science and Economy, University of Exeter, Penryn, UK

**Keywords:** social dynamics, kinship dynamics, *Orcinus orca*, life-history evolution, menopause

## Abstract

Age-related changes in the patterns of local relatedness (kinship dynamics) can be a significant selective force shaping the evolution of life history and social behaviour. In humans and some species of toothed whales, average female relatedness increases with age, which can select for a prolonged post-reproductive lifespan in older females due to both costs of reproductive conflict and benefits of late-life helping of kin. Killer whales (*Orcinus orca*) provide a valuable system for exploring social dynamics related to such costs and benefits in a mammal with an extended post-reproductive female lifespan. We use more than 40 years of demographic and association data on the mammal-eating Bigg's killer whale to quantify how mother–offspring social relationships change with offspring age and identify opportunities for late-life helping and the potential for an intergenerational reproductive conflict. Our results suggest a high degree of male philopatry and female-biased budding dispersal in Bigg's killer whales, with some variability in the dispersal rate for both sexes. These patterns of dispersal provide opportunities for late-life helping particularly between mothers and their adult sons, while partly mitigating the costs of mother–daughter reproductive conflict. Our results provide an important step towards understanding why and how menopause has evolved in Bigg's killer whales.

## 1. Introduction

Temporal changes in an individual's social environment are probably a key driver of age-based variation in life history and social behaviour [1–3]. Dispersal from the natal group is a key factor driving temporal changes in social structure [4,5], resulting in changes in an individual's local relatedness over their lifetime (kinship dynamics) [6]. Kinship dynamics can influence the indirect fitness costs and benefits associated with performing social behaviours, which may change systematically as a function of age [7]. In most mammals, dispersal is male-biased [8] and, depending on the mating pattern, will lead to females having constant or decreasing average relatedness to their group [7]. Under such conditions, selection for helping will decrease with female age, as her inclusive fitness benefits of helping will decline with decreasing relatedness to her group [7]. In rare mammalian cases, however, including ancestral humans and killer whales (*Orcinus orca*), temporal changes shaped by dispersal and mating patterns (female-biased dispersal with local mating and bisexual philopatry with non-local mating, respectively) create a scenario where females become increasingly related to their group on average as they age [7,9]. This pattern of kinship dynamics is linked to the evolution of a long post-reproductive female lifespan [7,9]. An increase in local relatedness with age is predicted to drive a stronger selection for helping (behaviours that have a positive impact on the fitness of members of the local group) in older females and a stronger selection for competitive effort (e.g. over reproduction) in younger females [7,9]. Thus, examining how the temporal dynamics of social associations impact the opportunities for helping and harming throughout an individual's life is key to understanding how age-related changes in the social environment influence the evolution of life history and social behaviours [10].

Killer whales have been a key model system to investigate the mechanisms underlying the evolution of menopause, with particular emphasis on the fish-eating resident killer whales of the northeastern Pacific Ocean [7,11–16]. In this killer whale ecotype, dispersal of individual males or females is extremely rare [17], and instead, dispersal occurs in the form of matrilineal splitting (females split from their natal group with their descendants) [18], and mating typically occurs between individuals of different groups [19,20]. From a female's perspective, life begins in a group where she has a relatively high relatedness to other females, as they are her mother, sisters and other maternally related females. While her brothers and other maternally related males are in her group, her average relatedness to males is comparatively low as her father is not in her group. As she starts having offspring of her own, her sons and daughters will stay in her group replacing more distantly related males, and her average relatedness to local males will increase [12]. In line with kinship dynamics theory, older killer whale females are more likely to perform behaviours that provide survival benefits to their close kin, such as sharing prey with kin and leading the group [13,21] with their presence in a group increasing the survival of their offspring and grandoffspring [14,15]. Moreover, consistent with the prediction that younger females should invest more in competitive effort: in an intergenerational conflict over reproduction where female killer whales from two generations have offspring at the same time, calves from the older-generation female suffer from higher mortality risk [12]. The combination of these inclusive fitness benefits and costs of reproductive overlap, can help explain why a long post-reproductive female lifespan has evolved in resident killer whales [7].

Killer whales are an ecologically and behaviourally diverse species, with various populations (ecotypes) showing variation in social organization [19,22–25]. Owing to this diversity, it was unclear until recently whether a prolonged post-reproductive lifespan is unique to resident killer whales, or is a general trait that occurs in other killer whale ecotypes. A recent analysis of long-term data has demonstrated that mammal-eating Bigg's killer whales (also known as transients), despite being ecologically, socially and genetically distinct from resident killer whales [17,19,26,27], have a comparable extended post-reproductive lifespan where more than 30% of adult female years are being lived by post-reproductive females [28]. This is somewhat surprising given the difference in social structure between the two ecotypes. The mammal-eating Bigg's killer whales have apparent partial dispersal of males and females around the age of maturity with some offspring remaining in their mother's group while others disperse. This dispersal pattern is likely driven by the ecological constraints in group size linked to hunting and sharing of marine mammal prey [22,29,30]. However, it remains unclear how the social relationship between mother and offspring changes over time, and by effect, the opportunity for mothers to help their offspring with age.

Here, we quantify the temporal changes of social relationships in Bigg's killer whale mother–offspring dyads. Specifically, we examine the opportunities for females to gain inclusive fitness benefits from helping adult offspring, which provides insight into the potential role of kin selection in driving the evolution of a prolonged post-reproductive female lifespan in Bigg's killer whales.

## 2. Material and methods

### (a) Bigg's killer whales

Bigg's killer whales are a mammal-eating ecotype found in the northeast Pacific Ocean. Here, we study a population of Bigg's, the West Coast transients, that range throughout coastal waters from southeastern Alaska to northern California [22,31] and are sympatric with populations of two other killer whale ecotypes: resident and offshore killer whales [22,31]. Although the three ecotypes have overlapping ranges, they are distinguished by their ranging patterns, where West Coast transient and resident killer whales typically occur in near-coastal waters compared to offshore killer whales that are a pelagic ecotype found mainly near the shelf-break [17,32]. Despite their overlap in range, Bigg's killer whales are reproductively isolated from these two ecotypes and genetic evidence indicates that they should be classified as a separate subspecies from resident and offshore killer whales [27]. Similar to resident killer whales, Bigg's killer whales live in groups of maternally related individuals, where females stop reproducing when they are in their late 30s or early 40s and can live for several decades following the onset of menopause [11,28,33]. Temporary and sometimes permanent dispersal from their maternal groups has been described for both sons and daughters in Bigg's killer whales, contrasting the stable social structure of resident killer whales where dispersal is rare by comparison [29–31,34–36]. The smaller group size of Bigg's killer whales compared to resident killer whales (resident group size of typically more than eight individuals [18,37]) has been linked to their main prey, pinnipeds and small cetaceans [30]. It should be noted that the average group size in Bigg's killer whales has increased since the beginning of the study period from 4.4 to 6.1 (mean minimum group size), overlapping with factors such as increased prey availability, population size increase and increased presence of the ecotype in the study area [34,36].

### (b) Data collection

The collection of photo-identification and life-history data on Bigg's killer whales was carried out between Alaska and California from 1972 to 2020. Bigg's killer whales can be observed in this area of their range during all months of the year, but with varying abundance, as some family groups are more resident to specific regions than others [22,36]. Identification photographs across the range and years were collected by various research organizations with central data repositories at the Center for Whale Research (CWR) in the USA and Fisheries and Oceans Canada (DFO). Photographs of the dorsal fin, saddle patches and eyepatches of the whales were taken during encounters with individuals or social groups. Whales were identified based on the shape of the dorsal fin as well as their saddle patch, unique markings, and/or scarring [22]. The sex of an individual was determined either genetically or visually based on morphological differences between the sexes as well as genital pigmentation on their posterior ventral surface [22]. Year of birth was determined based on the body size on the first observation for calves born between 1972 and 2020 and as the mean of potential years of birth for juveniles and young adults where there were missing observations of their mother for consecutive years overlapping their birth [22]. Birth year estimates for individuals born before the start of the study are conservative and are estimated for males from the year the dorsal fin began to sprout (rapid growth from age approx. eight to sexual maturity at age approx. 15) or was fully developed (physical maturity at age approx. 21) or for females using the year of the first known calf [22,38]. The year of death was either determined by strandings or if individuals were not sighted, either alone or in a group, on several occasions while their regular associates were sighted over several months or years [22]. The maternal relationship was determined based on observed mother–calf interactions, with genetic verification in some cases [22]. As the maternal pedigrees are not complete, we only determined the maternal relationships between individuals to a relatedness of *r* > 0.25. Encounters were filtered to include those marked as ‘full encounters' (high confidence in having identified all individuals present) in the database for 2005–2020. In years before 2005, the ‘full encounters’ field was not yet implemented in the database, and therefore encounters carried out by researchers associated with DFO or CWR were included if the minimum estimated number of whales present was less or equal to the number of identified whales from the encounter. This filtering resulted in the inclusion of 1019 out of 1742 encounters before 2005 and 4080 out of 5693 encounters from 2005 to 2020 from the data collected by DFO. Combined with the additional data from CWR (645 encounters), this provides a total of 5744 encounters spanning 48 years for the analyses introduced below (for yearly distribution of observations, see electronic supplementary material, figure S2).

### (c) Temporal stability of mother–offspring associations

Across the 48 years of encounters, we calculated the annual simple ratio association indices (SRI), describing the probability of observing a pair of individuals together when at least one of them was observed. Specifically, for each pair of individuals, yearly SRIs were calculated as the number of days the pair was identified during the same encounter out of the number of days where it would be possible to encounter the pair together (both individuals were alive and either both or one of them were identified) [39]. The SRI is appropriate for associations defined based on group membership [40] and when it is not possible to calibrate for detection biases before calculation [41].

To examine the stability of associations between observed mother–offspring dyads over time we calculated the lagged association rate (probability of two previously associating individuals being observed re-associated at different time lags) [42–44]. Associations among dyads of individuals can be based on spatial proximity, behavioural interactions or other criteria [45], and the thresholds used to define associations can have important impacts on the interpretation of social systems [46]. Owing to a lack of fine-scale resolution of social interactions for most of the encounters in our dataset and a general small group size of four to six individuals [36,47], whales observed together during the same encounter were defined as being part of the same group, and all individuals identified in the same group were assumed as associating equally. Potential dyads between individuals were only included in the analysis if both individuals were known to be alive in a given year. The lagged association rate was used to examine the temporal trends and persistence of associations over time [43,44]. The full dataset was used to analyse the lagged association to avoid a bias towards more stable and prevalent associations. Associations between mother and offspring were divided into juvenile and adult offspring based on age (adult female: more than 12 years, adult male: more than 14 years). The precision of the lagged association rates was evaluated using jackknife resampling [48].

### (d) Association categories

**Figure 1.**
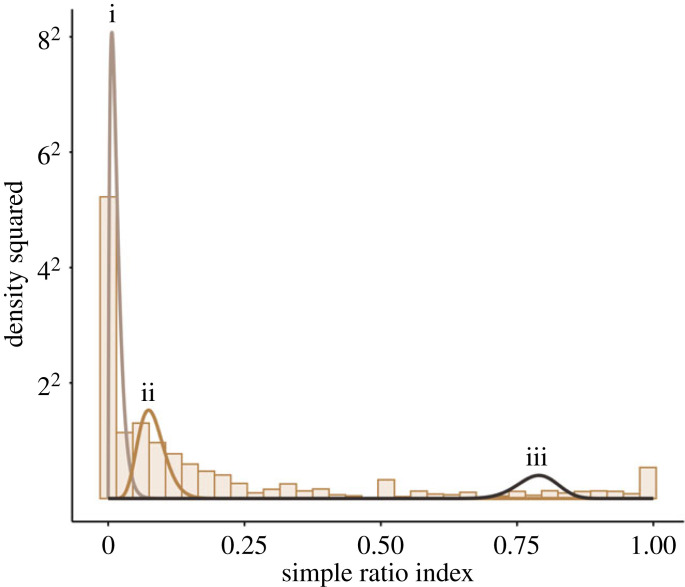
Density histogram plot of the observed simple ratio indices in 2020 (bars) with overlay of the binomial mixture model output for each of the three components (lines), with mean (*μ*) 0.0119 for distribution i (weak association), 0.1079 for distribution ii (casual acquaintance) and 0.9064 for distribution iii (constant companion).

The core social unit in Bigg's killer whales consists of a mother and her offspring (maternal group) [22,29–31]. Maternal groups are cohesive over long periods but will associate with other related and non-related maternal groups. The overall structure of observed association suggests that the social relationships fall into three types of associations: Constant companions (matriline/social unit members), casual acquaintances (preferred associates outside the core social unit), and weak associations (all other possible dyads in the population). We used a binomial mixture model approach [49,50] to investigate the number of social relationship categories in the association indices, which estimates the probability of a specific dyad being classified in each of the indicated association categories (see electronic supplementary material for full method description) [49,50]. A preliminary exploration of patterns in the associations using a binomial mixture model indicated that the distribution of social relationships falls into two to five relationship categories ranging from weak/absent to strong/constant. The qualitative pattern of especially the weakest and strongest social bonds identified by these models did not change substantially depending on the number of social relationship categories applied (electronic supplementary material, table S1 and figures S3 and S4). Using the information on social relationships from this preliminary examination as well as knowledge of the observed social organization of Bigg's killer whales, we applied a binomial mixture model using the *socmix* package in R [49–51] to categorize the observed association indices into three clusters representing three types of association in the population: constant companions, casual acquaintances and weak associations. This is supported by the distribution of the observed simple ratio indices into three distinct peaks matching the estimated beta-binomial distributions from the mixture model (figure 1).

### (e) Temporal dynamics of mother–offspring social relationships

To estimate the probability of each association belonging to any of the three components, the mixture model was applied for each year in the study period. For each association, the relationship bond was assigned based on the maternal pedigree (either through direct observations of mother–calf associations or genetic evidence). We explored the effect of the offspring's age and sex on the probability of an association being classified in each of the three types of associations (weak association, casual acquaintance or constant companion). We did this using a Bayesian generalized additive mixed effects model with a categorical response variable, *R_i_*, for each year the mother–offspring pair was encountered, *i*, with a probability of *p_i_* of being in one of the three association categories (*K* = 1:3):

$$R_{i}∼\mathrm{categorical}(\;p_{i,K=1:3}),$$

$$\mathrm{logit}(\;p_{i,K})=\beta_{s_{o,K}}+f_{s_{o,K}}(a_{i,K})+\varepsilon_{i,K},$$

$$\varepsilon_{i,K}∼N(0,\sigma_{\varepsilon }),$$

$$\sigma_{\varepsilon }∼t(0,2,3),$$

$$\beta_{s_{o,K}}∼N(0,2),$$

$$\mathrm{and}\;\sigma_{f}∼t(0,2,3).$$

Here, $\beta_{s_{o,K}}$ is an intercept specific to the sex of the offspring, while the term $\varepsilon_{i,K}$ is the individual-level random effect of the dyad. The term $f_{s_{o,K}}(a_{i,K})\;$represents the offspring sex and association category-specific smooth function over offspring age [52]. For the individual offspring where sex was unknown (*N* = 175), 100 imputations were performed to include uncertainty of sex for those individuals. The model was then run for all imputed datasets. The priors defined for intercept, error term, standard deviation and smooth function standard deviation were chosen to be weakly informative and were examined in prior predictive checks [53]. The model was fitted using an extended Hamiltonian Monte Carlo sampler (NUTS) [54] using the *brms* package in R via STAN [55]. We ran the model with four independent chains, 1500 warm-up iterations and 3000 iterations of sampling and evaluated that this produced an acceptable effective sample size to minimize uncertainty in estimates. The convergence of chains was checked by ensuring that $\hat{R}$ was less than 1.05 and by checking the trace plots for all runs. In an initial run of the model, there were divergent transitions and setting the delta step size to 0.99 eliminated those.

## 3. Results

### (a) Temporal stability of mother–offspring associations

**Figure 2.**
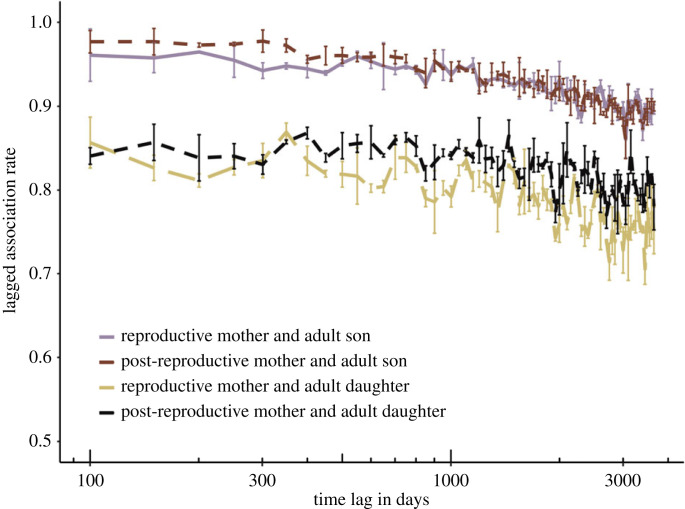
Lagged association rates over a maximum time lag of 10 years for associations between adult sons or daughters and their reproductive or post-reproductive mothers, including jackknife error bars. Note the limits of the *y*-axis.

The lagged association rate for mothers and their offspring remained consistently higher than for all other dyad types in the population (figure 2; electronic supplementary material, figure S1). Within mother–offspring associations, the association between mothers and their adult sons remained consistently higher than between mothers and their adult daughters, independent of the reproductive status of the mother (figure 2).

### (b) Association categories

**Figure 3.**
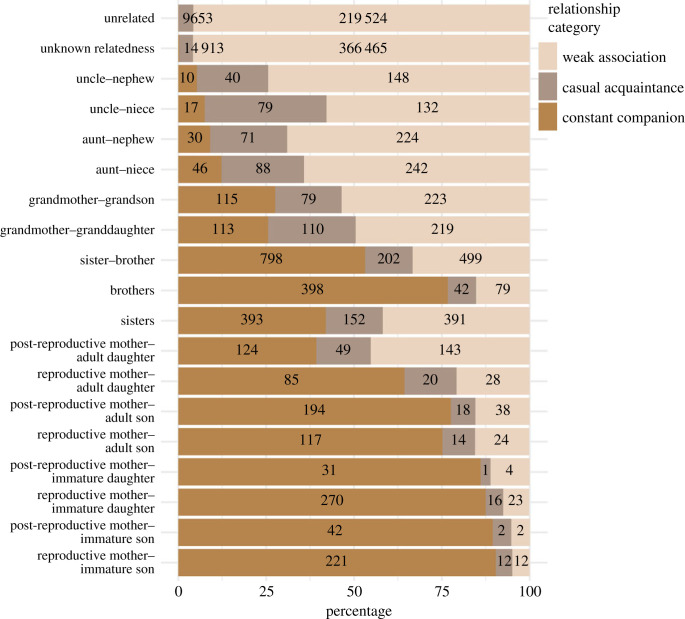
Stacked bar plot representing how many associations fall into each of the three relationship categories (weak association, casual acquaintances, and constant companion) for each type of dyad.

**Figure 4.**
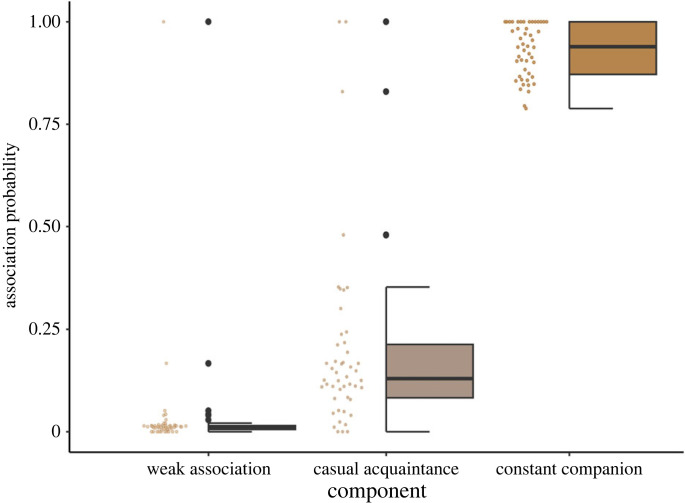
Boxplot representing the median (midline), 25th and 75th percentiles (lower and upper limits of the box, respectively), maximum and minimum of 1.5*inter-quartile range (upper and lower end of vertical lines, respectively) and outliers (black points) of the association probability from the binomial mixture model for the three components (weak association, casual acquaintance and constant companion) across the data period. Raw data is included for each boxplot as points.

The three-component mixture model for Bigg's killer whales included 478 individuals, 81 991 unique observed association pairs, and a total of 491 909 pairwise associations over 48 years. The bulk of these pairwise associations (467 005 or 95%) were classified as weak associations and typically occurred between maternally unrelated individuals (figure 3) with a mean ± s.d. association probability of 0.01 ± 0.06 (figure 4). A total of 18 622 (4%) were classified as a casual acquaintance with a mean ± s.d. association probability of 0.2 ± 0.1 (figure 4) and included associations between both related and maternally unrelated individuals (figure 3). Finally, 6282 associations (1.0%) were classified as a constant companion with a mean ± s.d. association probability of 0.9 ± 0.1 (figure 4), which were typically associations between mother and offspring (figures 3 and 4). Each year, a Bigg's killer whale has a mean ± s.d. of 3 ± 2 associations classified as a constant companion, 12 ± 12 as a casual acquaintance and 178 ± 74 as weak associations (note that weak association covers all possible associations in the population, including those that never occur). The majority of the potential dyads were non-relatives (47 625 dyads or 58%) or individuals of unknown maternal kinship (32 436 dyads or 40%). Maternal kinship to the second degree was known for 1930 (2%) of the possible annual dyads. As dyads were often observed in more than a single year there were a total of 12 139 associations between individuals of known relatedness to the second or first degree (e.g. mother–offspring, grandmother–grandoffspring).

### (c) Temporal dynamics of mother–offspring social relationships

**Figure 5.**
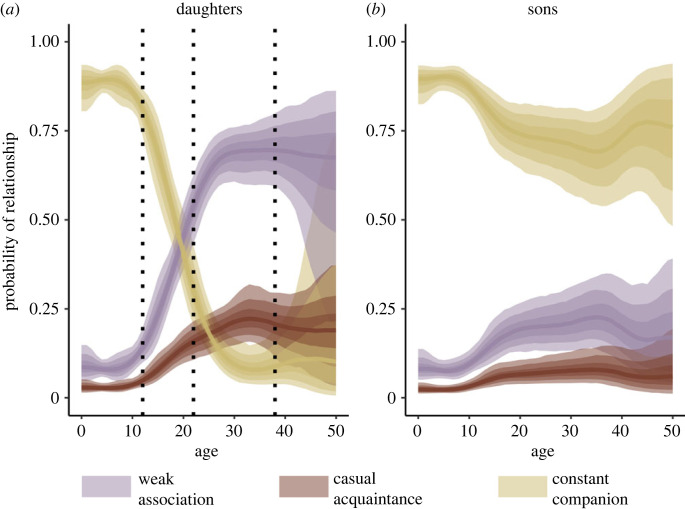
The probability of a mother–offspring association being classified in each of the three components (weak association, purple; casual acquaintance, rust; constant companion, yellow) with offspring age for (*a*) daughters and (*b*) sons. Lines represent the mean of the posterior distribution at each age and the shaded areas represents from darker to lighter the 50, 80 and 95% credible intervals, respectively. Vertical dotted lines in the plot (*a*) represent the ages at which 5, 50 and 95% of the female reproductive lifespan is estimated to have occurred [28].

In the 48 years of data, 390 unique mother–offspring dyads were observed, including 136 mothers, 115 sons, 132 daughters and 143 offspring of unknown sex. This sums up to 3747 association years, where a dyad observed associating in 10 different years equals 10 association years. Sons had a mean ± s.d. age of 17 ± 11 years, daughters had a mean age of 16 ± 10 years and individuals of unknown sex had a mean age of 5 ± 4 years. The pattern of change in associations between mother and offspring was dependent on both the sex and age of the offspring (figure 5). Sons had a gradual decline in the probability of having their mother as a constant companion with increasing age of the son but the mean remained high (greater than 0.75) even for older ages (figure 5*b*), while daughters, on the other hand, had a pronounced change in their bond to their mother with age—the probability of having their mother as a constant companion declined with increasing daughter age until age approximately 38 (figure 5*a*). For daughters, this change in association pattern is related to an increase in the probability of having a weak association with their mother, as well as the probability of having a casual acquaintance association with their mother (figure 5*a*). Sons on the other hand had a slight increase in the probability of having a weak association with their mother over time, while the probability of being a casual acquaintance remained low (figure 5*b*).

## 4. Discussion

Understanding the temporal dynamics of social associations is key to unlocking how age-related changes in the social environment influence selection for life-history traits and social behaviours. Here we show that social associations between mother and offspring in a long-lived social mammal are dynamic across the life of the offspring, likely influencing the opportunity for mothers and offspring to help or compete with close kin as a function of age. Specifically, social relationships between mothers and their offspring depend on the age and sex of the offspring, leading to mothers maintaining a stronger bond with sons compared to daughters in adulthood (figure 5). For Bigg's killer whales, the probability of having the strongest social bond with their mother decreases with the age for daughters but remains stable and high between mothers and their sons. Indeed, strong and stable bonds with sons are likely to provide the opportunity for mothers to perform helping behaviours that increase the survival of their sons and thus provide inclusive fitness benefits for the mother.

### (a) Temporal stability of mother–offspring associations

Killer whales are a highly social species. In the resident killer whale populations, both sons and daughters remain in their mother's group their entire lives leading to groups consisting of several generations of maternally related kin [19,23,56]. However, other killer whale populations appear to have a more fluid fission–fusion social structure [19,22,24,25]. Although previous research suggests that Bigg's killer whales have a similar fluid social structure [29], we show that across all associations between Bigg's mothers and their juvenile or adult offspring, the probability of re-associating remain very stable over a period of 10 years (figure 2 and electronic supplementary material, figure S1B). In other words, if offspring are associated with their mother into adulthood, they are likely to maintain the association for many years, potentially for life.

Although lagged association rates between a mother and a daughter and a mother and a son are both high and stable, there is a clear difference between associations with daughters compared to sons (figure 2). There is a higher probability of a mother re-associating with an adult son compared to an adult daughter, suggesting different patterns of associations depending on the sex of the offspring. Sex-biased associations could indicate that a daughter is more likely to become socially separate from their mother in adulthood compared with sons and that mothers may focus on opportunities to invest in adult sons. Mothers are likely to allocate more time and energy into the sex with the highest reproductive value, which can change given the age and sex of the offspring [57]. In resident killer whales, for example, adult sons have a higher reproductive potential, and offspring fathered by sons will likely be born outside of the group—thereby not introducing increased competition—making adult sons a favourable investment to increase inclusive fitness benefits for older mothers [7]. Indeed, resident killer whale mothers primarily share food with their adult sons [21] and if they lose their mother sons have an increased mortality risk [15]. This highlights the importance for resident mothers investing in the success of their sons, even when it is a substantial cost for herself [58]. Although it is not yet clear if similar benefits are present in Bigg's killer whales the stability of associations between mothers and sons suggests that Bigg's mothers could gain similar benefits. However, the lagged association rates are based on the observed association indices, which may be missing or underestimating important patterns of interactions between mother and offspring [59]. While future research is needed to quantify the fine-scale patterns of interaction between mothers and their offspring to determine the difference in maternal investment in sons versus daughters, our results show that daughters have a lower probability of remaining associated on a broader scale with their mother, supporting a sex-biased dispersal in this ecotype [29].

### (b) Temporal dynamics of mother–offspring social relationships

While mother–offspring associations remain stable for adult offspring over time independent of the sex of the offspring, there are clear differences in how social bonds between mother and offspring change depending on the age and sex of the offspring (figure 5). Mother–daughter dyads experience a gradual decrease in the probability of being in a constant companion association from the age of sexual maturity of the daughter, while mother–son dyads maintain an effectively constant probability of this strong relationship (figure 5). Thus, this pattern further supports a female-biased dispersal pattern with sons being fully or partially philopatric, as proposed by Baird & Whitehead [29].

The changes in mother–son associations, suggest that brothers may maintain stronger associations compared to sister–sister or sister–brother relationships (figure 3). This could be an effect of sons staying in close association with their mother and by exstension in close association with their brothers, while their sisters are more likely to disperse out of the group. Alternatively, this could be an effect of the subset of males that have been observed travelling outside of their mother's group, termed ‘roving' males, that might associate with a higher degree with their adult brothers, in comparison to their adult sisters. However, only eight males have been categorized as permanently ‘roving' while their mother was still alive, with the majority, therefore, having a high probability of maintaining a close association with their mother and by extension their brothers (electronic supplementary material, figure S5). More observations of male associations are needed to further investigate the dynamics of these relationships. As offspring reach ages above 40 years old, the credible intervals become very large, likely due to the sparsity of mother–offspring associations available in the data for offspring of greater than 40 years old.

#### (i) Group size and ecology

Offspring dispersal may help to maintain the foraging efficiency of the group [30,36]. Ecological considerations may therefore affect the dispersal of Bigg's killer whales as smaller groups could benefit from the increased energy efficiency from prey shared among group members, and increased hunting success due to a lower probability of alerting prey to their presence [30]. Within smaller family groups, cooperation and division of roles have been described, particularly for females [45]. Additionally, although observations of males actively taking part in hunts appear to be rare [60–62], the fact that they are likely to stay with their mother's group, suggests they also have the potential to provide a contribution to the group. Given the larger size of adult males, for example, they could benefit the group by taking extended dives preventing prey to escape [47] as well as during attacks of larger prey, such as sea lions or larger cetaceans [60–62]. With the advancements of observational methods, such as unmanned aerial systems, future studies should focus on the sex-specific division of roles during foraging to better understand the drivers of group composition in mammal-eating killer whales.

Across the 50 years of data, the observed average group size of Bigg's killer whales in the Salish Sea has increased from 4.4 to 6.1 individuals [35,36,47], coinciding with several factors: an increase in the availability of Bigg's prey [63–66], a rapid increase in the population size in the last approximately 30 years [34,67] and an increased presence of the Bigg's ecotype in the Salish Sea [36]. These factors appear to be related in that recovering prey populations correspond with an increase in available energy intake per individual, which subsequently drives increased group size, reproduction rates, overall abundance, and range use in Bigg's killer whales. It is likely that the advantages or costs of different dispersal tactics are dynamic and can change in response to ecological factors such as prey availability and a subsequent increase in population abundance. Additionally, factors relating to the individual offspring and the composition of the group, including birth order, number of siblings in the group and the reproductive state of the mother could all influence the balance of whether to stay or leave [18]. For example, depending on the age of the mother at the birth of the offspring, she may have reached menopause when the offspring becomes sexually mature, which could impact the benefits of staying or dispersing in comparison to an older sibling. From our results, there is a clear indication that daughters are more likely to disperse compared to sons and that this is true regardless of birth order (electronic supplementary material, figures S6 and S7). Future research should focus on teasing apart potential factors influencing the timing of dispersal to better understand how changes in the environment and group composition might impact the social structure of this ecotype.

#### (ii) Mothers, daughters and menopause

By maintaining a close relationship to their mother, adult females have the opportunity to benefit from the help of their mother and their siblings in the social group, probably receiving help via food sharing and alloparental care [21,68,69], while their mother may simultaneously benefit from help from adult offspring [70]. Additionally, a close association with their mother is also likely to be key for both sons and daughters to establish social bonds with kin and in gaining important skills via social learning from both mother and older siblings [71–73]. However, reproductive mothers and adult daughters that are in close association may compete over resources for reproduction. A mounting body of research has now shown that the age-linked changes in relatedness to group-mates—kinship dynamics—are integral in understanding the changes in selection for intergenerational helping and harming in females through their life [7,9]. In menopausal species, demographic patterns cause an asymmetry in the average relatedness to the group with age for females of different ages—leading to weaker selection for investment in reproductive competition in older females as intergenerational reproductive conflict would be more costly for older compared with younger females [7]. For example, when dispersal is female-biased or both sexes are philpatric and mating occurs outside of the group, females become increasingly related to their group as they age [7,9]. As a consequence, older females are under stronger selection for helping, while younger females are predicted to invest more in competitive effort compared to older females [7,12]. In resident killer whales, for instance, where both sexes are philopatric, the cost of conflict over reproduction is higher for older-generation females with a 1.7-time increase in offspring mortality risk for calves of older-generation mothers born into conflict [12]. Similarly in humans, reproductive conflict between different generation females have been shown to intensify the cost of reproduction for older females, selecting for females stopping reproduction early [9,74,75]. Even given the female-biased dispersal in Bigg's killer whales, for mothers and daughters the opportunity for a reproductive overlap remains as the decrease in the probability of being in the strongest association is gradual from the time daughters become sexually mature (the age when 5% of fecundity is expected to have occurred [28]) (figure 5*a*). More specifically, at the age when 50% of a Bigg's killer whale female fecundity is expected to have occurred the probability of being in the strongest relationship with their mother is greater than 0.25 [28] (figure 5*a*). However, unlike the resident killer whales, where daughters stay with their mother for their entire life, dispersal may have evolved in Bigg's killer whales as an additional mechanism to reduce reproductive overlap. Thus, the combination of female-biased dispersal and menopause likely results in a female reproductive overlap that is as small in Bigg's killer whales as it is in humans [9,74]. In other words, a daughter likely remains with her mother past the birth of at least her first offspring meaning that some level of intergenerational conflict (when mothers and the daughters co-breed) could occur. This pattern of kinship dynamics alone, however, is not sufficient to explain the presence of menopause. For example, females of both chimpanzees (*Pan troglodytes*) and banded mongoose (*Mungos mungo*) experience a similar increase in local relatedness with age, in addition to having overlapping reproduction between female generations [76]. Yet, neither have evolved menopause. A likely key difference between these species and those that have evolved menopause is the extent of the inclusive fitness effects of both late-life helping and intergenerational reproductive conflict [77]. Thus, quantifying such effects of late-life benefits of helping or costs of co-breeding between mother and daughter in Bigg's killer whales will provide valuable insight into the evolution of menopause in this killer whale ecotype.

Unlike other female-biased dispersers, such as chimpanzees, Bigg's killer whale females decrease the strength of association to their mother gradually from age of maturity and typically disperse after having at least their first offspring. This leads to a budding-event dispersal of a female and her offspring rather than individual dispersal prior to first reproduction [78,79]. Previous theoretical models of the kinship dynamics of dispersal by budding (concomitant dispersal of related individuals) have tended to focus on group-level rather than individual-level changes to average relatedness [6]. Additionally, dispersal has been modelled as individuals of the same cohort dispersing and establishing a new group [80,81], rather than a form of matrilineal budding of a female and her dependent offspring. In the latter case, a female would likely increase her average relatedness to her group at the time of dispersal, as she establishes a new group consisting of her and her exsisting offspring, thereby increasing her average relatedness to the group across her reproductively active period. Although the birth of grandoffspring will lower her average relatedness to her group, it may again increase following a budding event of a daughter and the daughter's offspring (grandoffspring). Thus, budding dispersal will impact the average relatedness of both the female that disperses as well as individuals in the group she dispersed from, including her mother. Future modelling should focus on exploring the individual-level changes to female average relatedness under such dispersal patterns to better understand the selection for intergenerational helping and competion under such social conditions.

The mating strategy of Bigg's killer whales is still unknown [67]. Yet, the high relatedness between group members suggests that mating occurs with individuals outside of the group perhaps as a mechanism of inbreeding avoidance, similar to resident killer whales [82], although both within-pod and within-matriline mating occurs in the southern resident killer whale population [20]. Despite the decrease in the probability of a mother and daughter being constant companions as the daughter matures, our results clearly show that Bigg's females have the opportunity to help kin, and especially sons, potentially through means of experience, ecological knowledge, leadership and food sharing, as seen in other mammals with long-lasting social relationships [13,21,83–85]. In addition, the gradual decline in the probability of being in a constant companion association with a daughter allows for a reproductive overlap between mother and daughter that could potentially lead to a comparable reproductive conflict as in other mammals with a long post-reproductive female lifespan [9,16]. Both of these mechanisms have been shown to influence the evolution of menopause in resident killer whales and could help to explain why it is also observed in Bigg's killer whales [12].

Here we have shown that social relationships between a Bigg's killer whale mother and her offspring depend on both the age and sex of the offspring, reflecting different changes in the social environment for males and females across their lifespan. In particular, for Bigg's females, the social environment changes throughout their lives, likely altering their average relatedness to their group and thus their inclusive fitness benefits and costs of performing both helping behaviours and competitive efforts. Specifically, Bigg's killer whale females experience an extended period of close association with adult offspring, particularly adult sons. During this period, they have the opportunity to gain the inclusive fitness benefits of helping, while also being in reproductive conflict with adult daughters, both of which could support the evolution of a long post-reproductive period in female Bigg's killer whales.

## Data Availability

Data available from the Dryad Digital Repository (https://doi.org/10.5061/dryad.n02v6wx25) [86]. For access to the database, contact Fisheries and Oceans Canada or Center for Whale Research. The data are provided in electronic supplementary material [87].
